# Chronic Lung Allograft Dysfunction in Patients Receiving Lung Transplantation for COVID-19 ARDS

**DOI:** 10.3389/ti.2025.14848

**Published:** 2025-11-04

**Authors:** Benjamin Thomae, Taisuke Kaiho, Austin Chang, Yudai Miyashita, Takahide Toyoda, Ambalavan Arunachalam, Ankit Bharat, G. R. Scott Budinger, Chitaru Kurihara

**Affiliations:** ^1^ Division of Thoracic Surgery, Department of Surgery, Northwestern University Feinberg School of Medicine, Chicago, IL, United States; ^2^ Division of Pulmonary and Critical Care Medicine, Department of Medicine, Northwestern University Feinberg School of Medicine, Chicago, IL, United States

**Keywords:** lung transplant, chronic lung allograft dysfunction (CLAD), COVID-19, acute respiratory distress syndrome, primary graft dysfunction

Dear Editors,

During the COVID-19 pandemic, lung transplant eligibility criteria were expanded to include patients with COVID-associated acute respiratory distress syndrome (CARDS) and post-COVID pulmonary fibrosis (PCPF). CARDS carries high mortality and often requires prolonged extracorporeal membrane oxygenation (ECMO) support and extended intensive care [1]. We previously reported the feasibility of lung transplantation in CARDS recipients, showing early survival comparable to non-CARDS recipients despite higher primary graft dysfunction (PGD) rates [2–6].

Long-term graft assessment in CARDS is essential to guide treatment and optimize resource use. Chronic lung allograft dysfunction (CLAD)—encompassing bronchiolitis obliterans syndrome (BOS), restrictive allograft syndrome (RAS), mixed, and undefined phenotypes—is the leading cause of late mortality [7]. Defined as sustained spirometric decline, CLAD affects approximately 30% of recipients within 3 years [8]. The incidence following CARDS remains unknown. This study evaluates long-term outcomes and the association between PGD and CLAD in CARDS recipients.

We conducted a single-center retrospective study of adult lung transplant recipients from January 2018 to December 2022. Patients were excluded if they died within 1 year, underwent multiorgan or repeat transplantation, or lacked sufficient spirometry (Supplementary Figure 1). Twenty-nine recipients which died within the first year (CARDS: 6; non-CARDS: 23) were excluded.

The primary outcome was CLAD incidence. Secondary outcomes included perioperative outcomes and multivariable CLAD predictors. PGD and CLAD were assessed by a multidisciplinary transplant team according to standard definitions [8]. CARDS transplant referrals followed our prior criteria (Supplemental Methods) [2–4].

A total of 252 patients were analyzed: 36 (14%) CARDS and 216 (86%) non-CARDS. Non-CARDS indications comprised interstitial lung disease (43%), chronic obstructive pulmonary disease (21%), pulmonary Artery Hypertension (8%), and others (28%). Compared with non-CARDS, CARDS recipients were younger (52.4 vs. 59.3 years; p = 0.002), less often smokers (19% vs. 53%; p < 0.001), more frequently bridged with venovenous (VV) ECMO use (50% vs. 4%; p < 0.001), had lower hemoglobin (9.1 vs. 12.0 g/dL; p < 0.001), and more often underwent bilateral lung transplantation (94% vs. 58%; p < 0.001). Median time from disease onset to listing was 104 days [IQR: 85–170]. Three-year survival was 79.8% overall and did not differ significantly between CARDS and non-CARDS (87.0% vs. 78.6%; HR 0.65, 95% CI 0.22–1.91; p = 0.17; Supplementary Figure 2) after adjustment for bilateral versus unilateral transplantation. Donor characteristics, including age, gender, and cause of death were comparable between groups and not associated with CLAD (Table 1).

**TABLE 1:** Characteristics and outcomes of patients and multivariate cox proportional hazards regression analysis to predict CLAD.

Variable	No CARDS (n = 216)	CARDS (n = 36)	P Value
Recipient factors			
Age, years	59.3 ± 12.4	52.4 ± 10.8	0.002
Female	88 (40.7%)	16 (44.4%)	0.72
Body Mass Index, kg/m^2^	25.8 ± 4.5	26.2 ± 4.6	0.56
Smoking history	116 (53.7%)	7 (19.4%)	<0.001
Hypertension	112 (51.9%)	16 (44.4%)	0.47
Diabetes	64 (29.6%)	12 (33.3%)	0.70
Chronic Kidney Disease	12 (5.6%)	0 (0%)	0.23
Pre-operative ECMO use	9 (4.2%)	18 (50.0%)	<0.001
Bilateral Transplantation	126 (58.3%)	34 (94.4%)	<0.001
Lung Allocation Score	50.3 ± 15.8	77.9 ± 16.4	<0.001
Follow-Up Days	808 [538–1,327]	1,079 [935–1,208]	0.02
Laboratory Values			
Hemoglobin, g/dL	12.0 ± 2.4	9.1 ± 1.9	<0.001
BUN, mg/dL	16.0 ± 6.0	20.3 ± 12.8	0.001
Creatinine, mg/dL	0.80 ± 0.22	0.60 ± 0.21	<0.001
PRA	85 (39.4%)	18 (50.0%)	0.27
Donor-specific antibodies	20 (9.3%)	9 (25.0%)	0.007
Donor			
Age, years	32.6 ± 11.8	30.8 ± 12.5	0.41
Female	65 (30.1%)	14 (38.9%)	0.33
Donor cause of death			
Head trauma	84 (38.9%)	17 (47.2%)	0.36
Anoxia	81 (37.5%)	16 (44.5%)	0.46
Other	51 (23.6%)	3 (8.3%)	0.05
Intra-operative outcomes			
Operative time (hours)	5.8 (4.8–7.5)	8.2 (7.4–9.5)	<0.001
Intra-op blood transfusion; pRBC	0 (0–2)	6 (2–11)	<0.001
Ischemic time (hours)	4.9 (4.1–5.8)	5.6 (5.1–6.0)	0.001
VA ECMO use	123 (56.9%)	34 (94.4%)	<0.001
VA ECMO time (hours)	1.7 (0–3.0)	3.1 (2.6–3.6)	<0.001
Postoperative outcomes – Univariate Analysis			
PGD	82 (38.0%)	21 (58.3%)	0.03
PGD Grade 3	14 (6.5%)	7 (19.4%)	0.02
Acute rejection	58 (38.9%)	2 (6.1%)	<0.001
post ECMO use	8 (3.7%)	13 (36.1%)	<0.001
Acute Kidney Injury	79 (36.6%)	18 (50.0%)	0.14
PE	7 (3.2%)	0 (0%)	0.60
Dialysis	17 (7.9%)	8 (22.2%)	0.01
CMV infection	15 (10.1%)	6 (18.2%)	0.23
ICU stay (days)	7 (5–11)	16 (10–22)	<0.001
Post-transplant ventilator (days)	2 (1–3)	4 (2–17)	<0.001
Hospital stay (days)	15 (11–27)	23 (17–37)	<0.001
Chronic Lung Allograft Dysfunction	46 (21.3%)	8 (22.2%)	1.00
BOS	36 (78.3%)	4 (50.0%)	0.18
RAS	6 (13.0%)	1 (12.5%)	1.00
Mixed	3 (6.5%)	3 (37.5%)	0.04
Undefined	1 (2.2%)	0 (0%)	1.00
Multivariable Analysis*	HR	P value	95% CI
Recipient Factors			
Body Mass Index, kg/m^2^	1.07	0.03	1.01–1.14
Lung Allocation Score	0.99	0.18	0.97–1.01
Hemoglobin, g/dL	1.06	0.41	0.93–1.20

Continuous data are shown as means ± standard deviation (SD) for age and laboratory data, and as medians and interquartile ranges [Q1-Q3] for days. *Variables with biological plausibility and a p-value <0.10 on univariate analysis were included in multivariable analysis.

CARDS recipients had longer operative (8.2 vs. 5.8 h; p < 0.001) and ischemic times (5.6 vs. 4.9 h; p < 0.001), more intraoperative VA-ECMO (94% vs. 56%; p < 0.001), greater blood transfusion (p < 0.001), higher PGD Grade 3 (19% vs. 7%; p = 0.02), and more dialysis (22% vs. 8%; p = 0.01). They also required longer ventilation (median 4 vs. 2 days; p < 0.001), ICU stays (16 vs. 7 days; p < 0.001), and hospitalization (23 vs. 15 days; p < 0.001) (Table 1). CLAD incidence was similar: 22% in CARDS (8/36) and 21% in non-CARDS (46/216; p = 1.00). The mixed phenotype was more common in CARDS with CLAD (38% vs. 7%; p = 0.04, possibly reflecting distinct immune activation or airway injury patterns after severe viral ARDS. In multivariable models, only BMI predicted CLAD (HR 1.07, CI 1.01–1.14; p = 0.03). Donor-specific antibodies, CMV infection, acute rejection, and transplant type were not significant predictors (Supplementary Table 1).

Although the coronavirus pandemic has subsided, lung transplantation remains a salvage option for patients with COVID–related respiratory failure and is currently being studied as a treatment for ARDS [9]. In this single-center case series, we report a 22% incidence of CLAD in CARDS patients undergoing lung transplantation, with an average follow-up of 1,079 days. This rate is not significantly different from the incidence in patients transplanted for other indications within our cohort (21%, p = 1.00) or from the 30% incidence reported at 1,095 days in international data [8]. These findings highlight the potential for long-term graft sustainability in this population and provide valuable single-center evidence on the feasibility of lung transplantation for ARDS in the post-COVID era.

CARDS recipients demonstrated significantly higher rates of PGD grade 3 compared to non-CARDS recipients (58.3% vs. 38.0%, p = 0.02), a key risk factor for CLAD [10]. Interpretation of this finding is complex, as the acute manifestations of CARDS—including inflammation, endothelial dysfunction, and pulmonary edema—can require prolonged ECMO support and impact PGD diagnostic criteria. Elevated PGD rates in CARDS patients may reflect acute disease severity rather than the traditional PGD pathophysiology described in lung transplant recipients with more chronic disease. Notably, only BMI was a significant predictor of CLAD in our 252-patient cohort (HR 1.07, CI 1.01–1.14, p = 0.03). Known risk factors such as PGD grade 3 was not significant [7–10]. This may reflect the limited sample size and the time-dependent nature of CLAD.

This study has several limitations, including modest sample size and mid-term follow-up, as well as a higher incidence of bilateral lung transplants in CARDS patients, which is associated with a longer time to CLAD diagnosis by a median of 150 days. In addition, 29 patients which died within the first year were excluded from the CLAD analysis. While this approach was necessary to meet the diagnostic definition of CLAD, it introduces the possibility of selection bias. We also acknowledge that death represents a competing risk when evaluating CLAD incidence, which was not formally modeled in this study. CARDS recipients in our cohort were younger and overall healthier compared with typical lung transplant candidates, which could partly explain the comparable CLAD rates observed. Although our study focused on CARDS, these findings may have implications for other acute respiratory failure syndromes, such as influenza-related ARDS; however, further research is needed before generalizing these results to other indications. In summary, our findings suggest CARDS is not associated with increased CLAD risk, and long-term outcomes remain favorable. Multi-center studies with extended follow-up are warranted.

## Data Availability

The original contributions presented in the study are included in the article/Supplementary Material, further inquiries can be directed to the corresponding author.
