# Supplementary material for: Chronic Lung Allograft Dysfunction in Patients Receiving Lung Transplantation for COVID-19 ARDS
Source: Transpl Int. 2025 Nov 4;38:14848. doi: 10.3389/ti.2025.14848 (PMC12623261; doi:10.3389/ti.2025.14848)
Supplement: Supplementary file 1 [file Supplementaryfile1.docx]

**SUPPLEMENTAL METHODS**

*Study Design*

This is a cohort study of adult patients who underwent lung transplantation at a single institution between January 2018 and December 2022. Patients who died within one year, underwent a multiorgan transplantation, underwent re-transplantation for a prior transplant before our study’s start date, or had less than four forced expiratory volume in one second (FEV1) measurements, precluding CLAD diagnosis, were excluded from the study. Our primary outcome was CLAD incidence, which we hypothesized would be higher in the CARDS cohort than the non-CARDS cohort. Perioperative and long-term post-operative characteristics and outcomes, as well as univariate and multivariate logistic regressions to predict CLAD, were obtained to further characterize our hypothesis of CLAD onset. To better assess mortality, we performed a supplemental Kaplan-Meier analysis of survival and CLAD-free survival, including patients who died within the first post-operative year. This supplemental cohort’s demographics and perioperative characteristics are described in a supplemental analysis. Patient data were collected retrospectively using electronic medical records.

This study was conducted in accordance with the Declaration of Helsinki and approved by the Institutional Review Board (IRB) of Northwestern University (STU00207250 and STU00213616). As this study was retrospective, patient consent for data collection was waived by the institutional review board.

*Statistical Analysis*

Recipient and donor characteristics, preoperative laboratory values, and intra- and postoperative outcomes were compared in lung transplant patients, categorized as with or without CARDS. The Mann-Whitney U test and Student t-test were used to compare independent, continuous variables between the groups. The chi-squared test was used to compare categorical variables reported as numbers and percentages. Kaplan-Meier analysis and the Wilcoxon signed-rank test were used to compare survival and CLAD-free survival between the two patient cohorts. Similarly to our previous studies, we conducted a multivariate analysis for variables on the basis of biological plausibility and based on univariate analysis.^1-3^ Statistical significance was set at p < 0.05.

*Definition of complication*

**Primary graft dysfunction (PGD)**

PGD was defined based on the ISHLT guideline and graded by PaO2/FiO2 ratio as follows; Grade 1: PaO2/FiO2 ratio >300; Grade 2: PaO2/FiO2 ratio is 200-300; Grade 3: PaO2/FiO2 ratio <200.^4^ The use of venovenous or venoarterial ECMO on day 3 was classified as Grade 3. PGD definitions were obtained on post-operative day 3.

**Chronic Allograft Dysfunction (CLAD)**

CLAD was also defined according to ISHLT guidelines including progressive loss of FEV1 in comparison to post-transplant baseline, radiographic changes, exclusion of infection, and transbronchial biopsy histology when appropriate.^8^ CLAD diagnoses were determined by our multi-disciplinary lung transplantation team.

**Acute kidney injury (AKI)**

AKI was defined using the Risk, Failure, Loss of kidney function, and End-stage kidney disease classification.^5^

*Indications for transplantation in COVID-19-associated Acute Respiratory Distress Syndrome (CARDS)*

ARDS was defined by the Berlin Definition.^6^ A referral for lung transplantation was made when a multidisciplinary team concluded that there was no longitudinal evidence of lung recovery at least 4 to 6 weeks after the onset of CARDS, a criterion consistent with previous studies.^7-9^ The multidisciplinary team distinguished patients with COVID-associated respiratory failure as either fulminant CARDS or PCPF pathology – our study includes only those with CARDS pathology. Lung transplant selections and evaluations were performed according to the ISHLT guidelines.^10^ The broad transplant criteria for patients with CARDS included people age 70 or younger, two consecutive lower respiratory fluid polymerase chain reaction tests negative for SARS-CoV-2 (on a specimen obtained by endotracheal aspirate or bronchoscopy 24 hours apart), single organ failure, no evidence of irrecoverable brain damage, and a body mass index (BMI) less than or equal to 35. The same BMI cutoff was used for lung transplant recipients without COVID-19.^7^ Excluding the first patient in the CARDS cohort who lacked decisional capacity and was unable to participate, all CARDS patients consented to lung transplantation. Importantly, 34 of the 36 CARDS patients (94%) received bilateral lung transplantation.

**References**

1. Cerier, Emily, Adwaiy Manerikar, Viswajit Kandula, Takahide Toyoda, Benjamin Thomae, Yuriko Yagi, Diego Mauricio Avella Patino, et al. “Postreperfusion Pulmonary Artery Pressure Indicates Primary Graft Dysfunction After Lung Transplant.” *The Annals of Thoracic Surgery* 117, no. 1 (January 2024): 206–12. <https://doi.org/10.1016/j.athoracsur.2022.12.013>.
2. Kaihou, Taisuke, Takahide Toyoda, Emily Cerier, Yuriko Yagi, Adwaiy Manerikar, Benjamin Louis Thomae, Viswajit Kandula, Ankit Bharat, and Chitaru Kurihara. “The Risk of Pretransplant Blood Transfusion for Primary Graft Dysfunction After Lung Transplant.” *Annals of Thoracic Surgery Short Reports* 2, no. 3 (March 5, 2024): 573–77. <https://doi.org/10.1016/j.atssr.2024.02.004>.
3. Graham, Kahla, Taisuke Kaiho, Benjamin Louis Thomae, Yuriko Yagi, Emily Cerier, Bonnie Martin-Harris, Ankit Bharat, and Chitaru Kurihara. “Risk Factors and Impact of Swallowing Impairment and Aspiration after Lung Transplantation.” *Journal of Thoracic Disease* 16, no. 9 (September 30, 2024). <https://doi.org/10.21037/jtd-24-707>.
4. Snell GI, Yusen RD, Weill D, et al. Report of the ISHLT Working Group on Primary Lung Graft Dysfunction, part I: Definition and grading-A 2016 Consensus Group statement of the International Society for Heart and Lung Transplantation. *J Heart Lung Transplant*. 2017;36(10):1097-1103. doi:[10.1016/j.healun.2017.07.021](https://doi.org/10.1016/j.healun.2017.07.021)
5. Bellomo R, Ronco C, Kellum JA, Mehta RL, Palevsky P, Acute Dialysis Quality Initiative workgroup. Acute renal failure - definition, outcome measures, animal models, fluid therapy and information technology needs: the Second International Consensus Conference of the Acute Dialysis Quality Initiative (ADQI) Group. *Crit Care*. 2004;8(4):R204-212. doi:[10.1186/cc2872](https://doi.org/10.1186/cc2872)
6. ARDS Definition Task Force, Ranieri VM, Rubenfeld GD, et al. Acute respiratory distress syndrome: the Berlin Definition. *JAMA*. 2012;307(23):2526-2533. doi:[10.1001/jama.2012.5669](https://doi.org/10.1001/jama.2012.5669)
7. Kurihara C, Manerikar A, Querrey M, et al. Clinical Characteristics and Outcomes of Patients With COVID-19-Associated Acute Respiratory Distress Syndrome Who Underwent Lung Transplant. *JAMA*. 2022;327(7):652-661. doi:[10.1001/jama.2022.0204](https://doi.org/10.1001/jama.2022.0204)
8. Bharat A, Querrey M, Markov NS, et al. Lung transplantation for patients with severe COVID-19. *Sci Transl Med*. 2020;12(574):eabe4282. doi:[10.1126/scitranslmed.abe4282](https://doi.org/10.1126/scitranslmed.abe4282)
9. Bharat A, Machuca TN, Querrey M, et al. Early outcomes after lung transplantation for severe COVID-19: a series of the first consecutive cases from four countries. *Lancet Respir Med*. 2021;9(5):487-497. doi:[10.1016/S2213-2600(21)00077-1](https://doi.org/10.1016/S2213-2600(21)00077-1)
10. Leard LE, Holm AM, Valapour M, et al. Consensus document for the selection of lung transplant candidates: An update from the International Society for Heart and Lung Transplantation. *J Heart Lung Transplant*. 2021;40(11):1349-1379. doi:[10.1016/j.healun.2021.07.005](https://doi.org/10.1016/j.healun.2021.07.005)
