# Supplementary material for: Chronic Lung Allograft Dysfunction in Patients Receiving Lung Transplantation for COVID-19 ARDS
Source: Transpl Int. 2025 Nov 4;38:14848. doi: 10.3389/ti.2025.14848 (PMC12623261; doi:10.3389/ti.2025.14848)
Supplement: Supplementary file 2 [file Table1.docx]

**Supplemental Table 1. Univariate Cox Proportional Hazards Regression Analysis to Predict CLAD**

| Variable | HR | *P* value | 95% CI |
| --- | --- | --- | --- |
| Recipient factors |  |  |  |
| Age, years | 0.99 | 0.21 | 0.97-1.01 |
| Female | 0.82 | 0.48 | 0.47-1.42 |
| Body Mass Index, kg/m2 | 1.08 | 0.02 | 1.01-1.15 |
| Smoking history | 1.22 | 0.48 | 0.71-2.08 |
| Hypertension | 1.12 | 0.69 | 0.65-1.91 |
| Diabetes | 1.35 | 0.30 | 0.77-2.34 |
| Chronic Kidney Disease | 1.88 | 0.18 | 0.75-4.75 |
| Pre-operative ECMO use | 0.93 | 0.81 | 0.54-1.62 |
| Bilateral Transplantation | 0.83 | 0.52 | 0.48-1.45 |
| Lung Allocation Score | 0.98 | 0.04 | 0.97-1.00 |
| Etiology |  |  |  |
| CARDS | 0.89 | 0.76 | 0.42-1.90 |
| Interstitial Lung Disease | 1.07 | 0.82 | 0.61-1.86 |
| COPD | 1.07 | 0.86 | 0.53-2.12 |
| Pulmonary Artery Hypertension | 0.55 | 0.31 | 0.17-1.75 |
| Laboratory Values |  |  |  |
| Hemoglobin, g/dL | 1.11 | 0.05 | 0.99-1.24 |
| WBC, 1,000/mm3 | 0.99 | 0.71 | 0.92-1.06 |
| Platelets, 1,000/mm3 | 1.00 | 0.97 | 0.99-1.00 |
| BUN, mg/dL | 1.02 | 0.35 | 0.98-1.05 |
| Creatinine, mg/dL | 1.48 | 0.52 | 0.45-4.94 |
| PRA | 0.90 | 0.70 | 0.52-1.55 |
| Donor-specific antibodies | 1.46 | 0.20 | 0.82-2.61 |
| Intra-operative outcomes |  |  |  |
| Operative time (hours) | 1.00 | 0.80 | 0.99-1.01 |
| Intra-op blood transfusion; pRBC | 0.98 | 0.61 | 0.91-1.07 |
| Ischemic time (hours) | 1.00 | 0.91 | 0.99-1.01 |
| VA ECMO use | 0.94 | 0.81 | 0.54-1.62 |
| Postoperative outcomes |  |  |  |
| PGD | 0.80 | 0.44 | 0.46-1.41 |
| PGD grade3 | 0.79 | 0.65 | 0.29-2.20 |
| Acute rejection | 1.71 | 0.08 | 0.94-3.12 |
| post ECMO use | 0.92 | 0.86 | 0.37-2.32 |
| Acute Kidney Injury | 0.66 | 0.17 | 0.36-1.20 |
| PE | 1.41 | 0.64 | 0.34-5.83 |
| Dialysis | 0.89 | 0.81 | 0.35-2.24 |
| CMV infection | 1.27 | 0.59 | 0.54-3.02 |

CLAD, chronic lung allograft dysfunction; COPD, chronic obstructive pulmonary disease; BMI, body mass index; BSA, body surface area; ECMO, extracorporeal membrane oxygenation; LAS, lung allocation score; COVID-19, coronavirus disease 2019; PRA, panel reactive antibody; DSA, donor specific antibody; pRBC, packed red blood cells; FFP, fresh frozen plasma; Plt, platelets; VA-ECMO, veno-arterial extracorporeal membrane oxygenation; PGD, primary graft dysfunction; AKI, acute kidney dysfunction
