# Supplementary material for: Chronic Lung Allograft Dysfunction in Patients Receiving Lung Transplantation for COVID-19 ARDS
Source: Transpl Int. 2025 Nov 4;38:14848. doi: 10.3389/ti.2025.14848 (PMC12623261; doi:10.3389/ti.2025.14848)
Supplement: Supplementary file 3 [file DataSheet2.docx]

**Supplemental Figure 2.** Kaplan-Meier analysis of overall survival after lung transplantation for CARDS and non-CARDS patients.

**
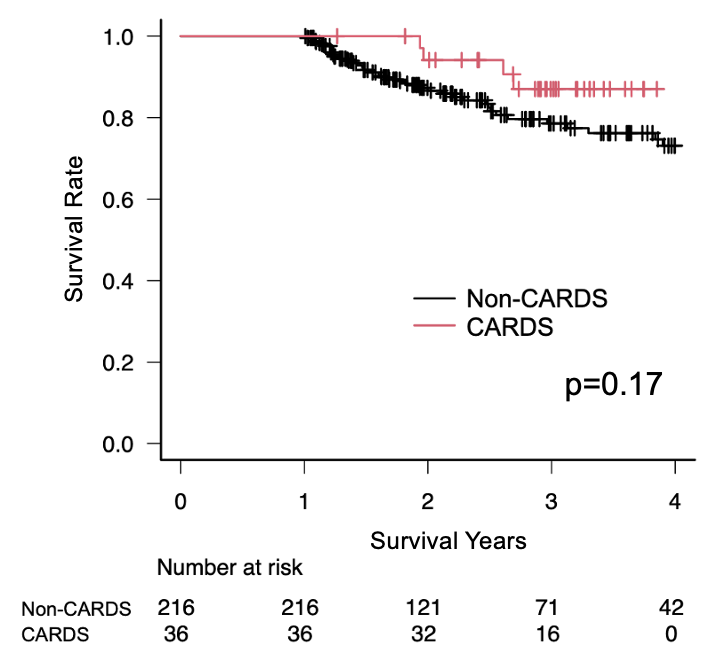
**
