# Supplementary figures and images for: Chronic Lung Allograft Dysfunction in Patients Receiving Lung Transplantation for COVID-19 ARDS
Source: Transpl Int. 2025 Nov 4;38:14848. doi: 10.3389/ti.2025.14848 (PMC12623261; doi:10.3389/ti.2025.14848)

**Supplemental Figure 1.** Study population

**
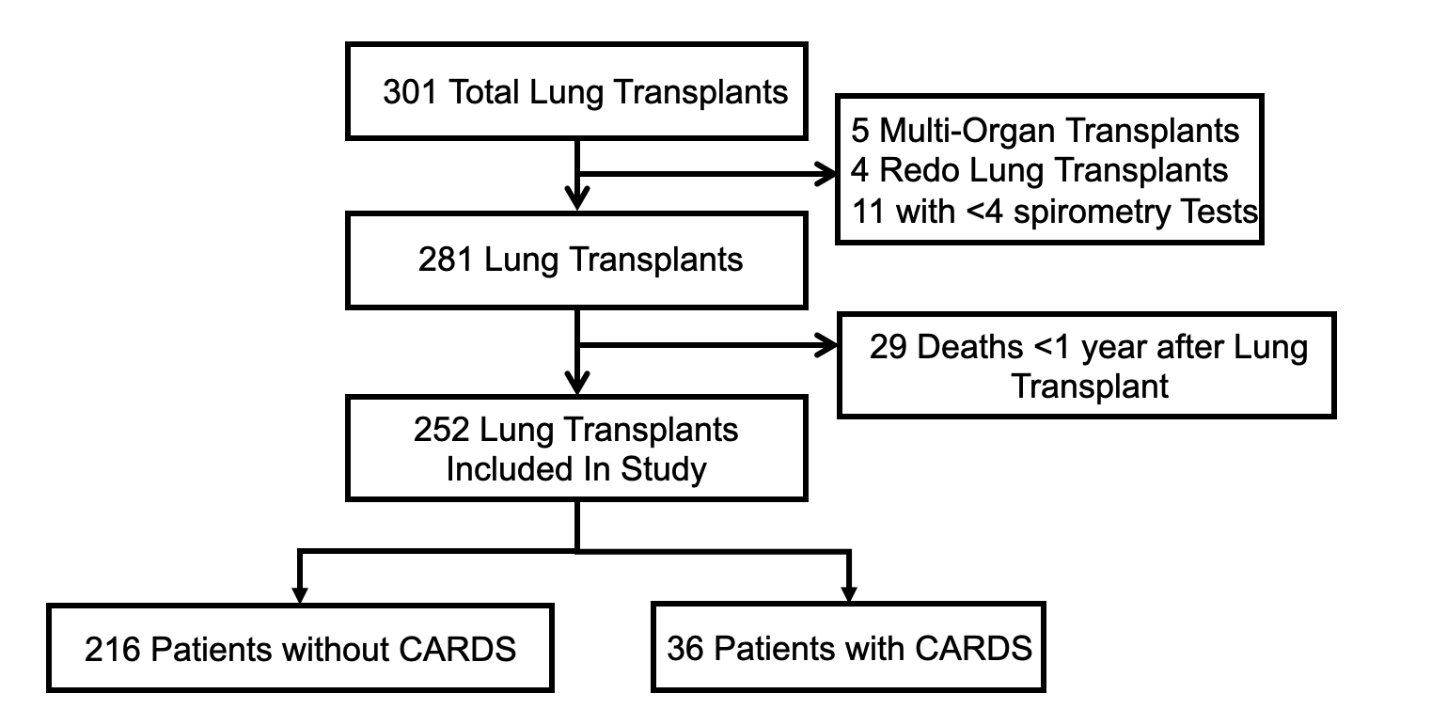
**

Supplement: Supplementary file 4 [file DataSheet1.docx]
